# Study of the Mechanical, Sound Absorption and Thermal Properties of Cellular Rubber Composites Filled with a Silica Nanofiller

**DOI:** 10.3390/ma14237450

**Published:** 2021-12-04

**Authors:** Marek Pöschl, Martin Vašina

**Affiliations:** 1 Centre of Polymer Systems, Tomas Bata University in Zlin, Třída Tomáše Bati 5678, 760 01 Zlin, Czech Republic; poschl@utb.cz; 2 Faculty of Technology, Tomas Bata University in Zlin, Nám. T.G. Masaryka 275, 760 01 Zlin, Czech Republic; 3 Faculty of Mechanical Engineering, VŠB-Technical University of Ostrava, 17. listopadu 15/2172, Poruba, 708 00 Ostrava, Czech Republic

**Keywords:** cellular rubber, mechanical stiffness, sound absorption, vibration damping, thermal behavior, silica nanofiller, excitation frequency

## Abstract

This paper deals with the study of cellular rubbers, which were filled with silica nanofiller in order to optimize the rubber properties for given purposes. The rubber composites were produced with different concentrations of silica nanofiller at the same blowing agent concentration. The mechanical, sound absorption and thermal properties of the investigated rubber composites were evaluated. It was found that the concentration of silica filler had a significant effect on the above-mentioned properties. It was detected that a higher concentration of silica nanofiller generally led to an increase in mechanical stiffness and thermal conductivity. Conversely, sound absorption and thermal degradation of the investigated rubber composites decreased with an increase in the filler concentration. It can be also concluded that the rubber composites containing higher concentrations of silica filler showed a higher stiffness to weight ratio, which is one of the great advantages of these materials. Based on the experimental data, it was possible to find a correlation between mechanical stiffness of the tested rubber specimens evaluated using conventional and vibroacoustic measurement techniques. In addition, this paper presents a new methodology to optimize the blowing and vulcanization processes of rubber samples during their production.

## 1. Introduction

Natural rubber (NR) comes from a tree called “*Hevea brasiliensis*” that is grown in many countries around the world, e.g., in Malaysia, Indonesia, India, Vietnam and Thailand. This tree produces a milky liquid called natural rubber latex [1]. Natural rubber latex is a dispersion of rubber particles in aqueous medium containing approximately 33% cis-1,4-polyisoprene rubber particles dispersed in water. This latex is obtained from the tree by making a cut in its bark in tropical countries of its origin [2]. The rubber is prepared by coagulation of the latex with acids. The obtained precipitate is subsequently dried and pressed into packaging. Natural rubber is very important in the rubber industry due to its elasticity, tensile strength and self-reinforcing character, which is said to originate from its strain-induced crystallization (SIC). SIC is caused by its linear chains, which are able to orient under stress. Rubbers with a macromolecular structure like polychloroprene rubber (CR) and polyisoprene rubber (IR) similar to NR can crystallize under stress. Due to the SIC behavior, these rubbers have excellent mechanical properties and good resistance to crack growth [3,4,5]. The advantages of NR also include low heat build-up [6]. 

However, unvulcanized rubbers without ingredients are not often used because they do not have the required properties. Additionally, additives (fillers, vulcanizing agents, UV stabilizers, etc.) are added to these rubbers to achieve the required properties. NR with additives is referred to as a rubber compound that is plastic and malleable. The composition of rubber compounds is given in units of phr (i.e., parts per hundred rubber). Crosslinking process occurs during high-temperature heating, when the rubber compound turns into an elastic material, i.e., vulcanizate [7,8]. Sulphur is one of the most commonly used curing agents [7]. Peroxides usually for ethylene propylene diene methylene (EPDM) and oxides of divalent metals for CR rubbers are also used. The main components of rubber compounds are fillers because they reduce the cost of rubber products and modify physical and mechanical properties of vulcanizates, such as tensile strength and abrasion [8]. The stiffening effect of rubber compounds generally depends on filler particle sizes. The highest stiffness is usually achieved with rubber compounds containing filler particle sizes from 10 to 100 nm that are characterized by a large specific surface area. In practice, carbon black and silica nanofillers are most commonly used as reinforcing fillers [9]. Silica is obtained from silica rocks by natural mining of minerals in various parts of the world and by the subsequent grinding or crushing of these raw materials. Such silica is called ground silica and is characterized by a low reinforcing effect due to the large primary particles. By chemical treatment of the mined material, it is possible to obtain silica with small particle sizes, which have a stiffening effect comparable to that of carbon black filler. The smallest primary particle sizes (i.e., from 1 to 3 nm) are obtained by pyrolysis process. Such silica is called fumed silica [10]. These primary particles form aggregates of size from 100 to 250 nm. Silica is characterized by the best stiffening effect due to its large specific surface area (i.e., from 50 to 400 m^2^·g^−1^) [11]. Larger particle sizes (>40 nm) are obtained by precipitation of sodium silicate (Na_2_SiO_3_) with acids. Such silica is called precipitated silica [10,12].

Other additives are also added to rubber compounds. Nucleating agents are used to improve the cell morphology of rubber compounds (i.e., increasing cell density, decreasing cell size and narrowing cell-size distribution) by providing heterogeneous nucleation sites [13]. Blowing agents are added in order to produce foam rubber products. Along with urethane and polyvinyl chloride rubber materials, the foam rubber products are competitive with cushioning or padding plastic foams such as cloth, polyester fiberfill, hair, jute and the like. Foam rubber products are very important in many applications due to their unique structural properties such as their low weight, performance, impact damping, thermal and acoustic insulation properties, moderate energy absorption and low price [14,15,16]. In general, foam rubbers can be divided into sponge, cellular and microcellular rubbers. Sponge rubber has a similar structure to a real sea sponge. It consists of open and interconnected cavities. The channels thus formed allow a high absorption of liquids and release the absorbed liquid after their compression. Cellular rubber is characterized by a higher specific weight compared to sponge rubber. Its structure, characterized by cell size, cell density and expansion ratio [17], consists of smaller cavities that are not interconnected. The cellular rubber is thus more compressible, non-absorbent and achieves excellent damping properties [14]. Microcellular rubbers are characterized by cell densities greater than 109 cells/cm^3^ and cells smaller than 10 μm [18]. These rubber types are produced by using physical or chemistry blowing agents. Physical blowing is performed with blowing liquids (e.g., pentane and hexane) and is based on the liquid–vapor transition. The essence of chemical blowing is the chemical decomposition of the blowing agent or the reaction to release gases. In the rubber blowing process, it is important that the blowing process takes place before vulcanization. Once the cellular structure is formed, vulcanization should occur to fix this cellular structure [14,19,20]. Another type of foam rubber is latex foam, which is obtained from natural rubber latex by mechanical whipping and subsequent vulcanization. New applications of the NR latex foam rubbers allow their use as absorbent materials for oils and carbon dioxide (CO_2_). NR latex foam rubber filled with silica can serve as a CO_2_ absorbing material [21]. 

Many studies have already investigated various properties of silica-filled rubber composites. The aim of this research is to investigate the mechanical, sound absorption and thermal properties of cellular rubber composites that were produced with various concentrations of silica nanofiller and the same blowing agent concentration. Mechanical properties of rubber materials can be determined by conventional measurement techniques such as tensile, shear, hardness, permanent deformation, rebound resilience and viscoelasticity tests. In this research, the mechanical stiffness of the investigated silica-filled rubber composites determined from permanent deformation and rebound resilience measurements is verified by non-conventional methods for determining the stiffness of these materials, namely by vibration damping and sound absorption methods. The latter two methods are relatively simple, inexpensive, fast and non-destructive compared to the conventional methods used to determine the mechanical stiffness of solids. Therefore, the vibration damping and sound absorption methods can also be easily applied to compare the mechanical stiffness of different materials. In addition, the rubber molding process is optimized using rubber processing analysis, where the rubber blowing phase should generally take place before the vulcanization phase for a given rubber recipe.

## 2. Materials and Methods

### 2.1. Materials

Natural rubber SMR (Standard Malaysian Rubber)–20 was used as the base for rubber compounds. Silica Perkasil KS–408 from WR Grace & Co. (Columbia, MD, USA) with the specific surface area of 175 m^2^·g^−1^ and bulk density of 0.17 g·cm^−3^ was used as a nanofiller in rubber compounds. The additives, including activator ZnO from SlovZink a.s. (Košeca, Slovakia), activator stearic acid from Setuza a.s. (Ústí nad Labem, Czech Republic), antiozonant N-(1,3-dimethylbutyl)-N’-phenyl-p-phenylenediamine (Vulkanox 4020) from Lanxess AG (Brussel, Belgium), plasticizer (paraffinic oil Nyflex 228) from Nynas AB (Stockholm, Sweden), blowing agent azodicarbonamide C_2_H_4_N_4_O_2_ (Porofor ADC) from Lanxess AG (Brussel, Belgium), accelerator N-tert-butyl-benzothiazole sulfonamide (TBBS) from Duslo a.s. (Šal’a, Slovakia) and curing agent sulphur type Crystex OT33 from Eastman Chemical company (Kingsport, TN, USA), were also compounded. Five rubber compounds with different silica concentrations were prepared. Their recipe is detailed in Table 1. 

The optimization of mechanical properties of rubber compounds is highly dependent on the silica concentration. During the mixing process of rubber compounds, the viscosity of these compounds generally increases with increasing the silica concentration. High concentrations of silica filler (i.e., over 30 phr) in rubber compounds lead to a difficult mixing process, a lower dispersion of additives and an increase in curing time [22]. For this reason, rubber samples produced with the highest silica concentration of 45 phr are characterized by an imperfect blowing process, which results in a reduction in damping properties of cellular rubbers. 

In the first stage of the production of rubber composites, the basic rubber compounds (i.e., SMR–20, ZnO, Vulkanox 4020, stearic acid, silica and Nyflex 228) were mixed in a two-roll mill (150 mm × 330 mm, Farrel, Milan, Italy) at a rotation speed ratio of 15/20 at a temperature of 50 °C. After mixing these ingredients, a three-minute homogenization took place on the two-roll mill and subsequently the compound was withdrawn from this mill. The mixing process in the second stage, in which the remaining ingredients (i.e., Porofor ADC, TBBS and sulphur OT33) were mixed, followed after a 20-min pause on the two-roll mill under the same conditions as in the first stage. After this mixing, a five-minute homogenization was performed, and the obtained rubber compounds were subsequently withdrawn from the mill.

The rubber compounds were compression-molded at a temperature of 170 °C using a hydraulic hot press during the time *t_m_*, which is given by the equation:

(1) $$t_{m}=t_{90}+c_{m}\cdot t$$

where *c_m_* = 0.5 min/mm is the molding constant, *t*_90_ (min) is the optimum cure time (see Chapter 3.1) and *t* (mm) is the sample thickness.

Finally, rubber specimens of two different diameters of 20 and 100 mm and three different thicknesses of 4, 8 and 12 mm were produced.

### 2.2. Measurement Methodology

#### 2.2.1. Curing Characteristics

Curing characteristics were measured according to ASTM D 5289 standard on the Premier MDR moving die rheometer from Alpha Technology (Hudson, OH, USA) at a constant temperature of 170 °C. Minimum (*M_L_*) and maximum (*M_H_*) torques, scorch (*t_s_*_1_) and optimum cure (*t*_90_) times were subsequently evaluated.

#### 2.2.2. Rubber Processing Analysis

The rubber processing (RP) analysis, which is based on cone-cone principle, was performed on the Premier RPA rubber process analyzer from Alpha Technology (Hudson, OH, USA). As a result of RP analysis, time dependencies of pressure curves during isothermal vulcanization were determined. The rate of blowing was subsequently obtained from the first time derivative of the pressure curve. 

#### 2.2.3. Optical Microscopy

Pore sizes on cut surfaces of the investigated rubber specimens were evaluated using an optical microscope “Carl Zeiss Stemi 2000–C” from Carl Zeiss Microimaging GmbH (Jena, Germany) [23]. The magnification of the microscope objective was from 0.65× to 5× and the stereo angle was set to 11°.

#### 2.2.4. Rebound Resilience

The rebound resilience was measured according to ISO 4662 standard. Rubber specimens measuring 12 mm in thickness were examined. Average values of the rebound resilience and their standard deviations were subsequently determined from six measured values. The measurements were carried out at an ambient temperature of 22 °C.

#### 2.2.5. Compression Testing

Experimental measurements of permanent deformation of cellular rubber composites were performed according to ČSN EN ISO 1856 standard based on compression tests. These measurements were carried out on rubber samples measuring 12 mm in thickness and 20 mm in diameter. The samples were compressed to 50% initial thickness (*t*_1_) for 24 h at three different temperatures (i.e., 24, 40 and 65 °C). After compression, the samples were released for 30 min, and their thicknesses (*t*_2_) were measured again. Finally, the compression set *CS* (%) of the rubber samples was determined from the equation [24]:

(2) $$CS=\frac{t_{1}-t_{2}}{t_{1}}\times 100$$

Average values of the compression set, including their standard deviations, were determined from five measured values.

#### 2.2.6. Mechanical Vibration Damping Testing

The ability of a material to dampen harmonically excited mechanical vibration is expressed by the displacement transmissibility *T_d_* (–) that is defined for a linear single degree of freedom as follows [25,26]:

(3) $$T_{d}=\frac{y_{2}}{y_{1}}=\frac{a_{2}}{a_{1}}=\sqrt{\frac{k^{2}+{\left(c\omega \right)}^{2}}{{\left(k-m\omega^{2}\right)}^{2}+{\left(c\omega \right)}^{2}}}=\sqrt{\frac{1+{\left(2\zeta r\right)}^{2}}{{\left(1-r^{2}\right)}^{2}+{\left(2\zeta r\right)}^{2}}}$$

where *y* is the displacement amplitude on the input (1) and output (2) sides of the harmonically loaded sample, *a* is the acceleration amplitude on input (1) and output (2) sides of the harmonically loaded sample, *k* is the stiffness (spring constant), *c* is the viscous damping coefficient, *m* is the mass, *ω* is the frequency of oscillation, *ζ* is the damping ratio and *r* is the frequency ratio. The damping and frequency ratios are expressed by the equations [27,28,29]:

(4) $$\zeta =\frac{c}{2\cdot \sqrt{k\cdot m}}$$

(5) $$r=\frac{\omega }{\omega_{n}}=\frac{\omega \cdot \sqrt{m}}{\sqrt{k}}$$

where *ω_n_* is the natural frequency. There are three different types of mechanical vibration depending on the displacement transmissibility value, namely damped (*T_d_* < 1), undamped (*T_d_* = 1) and resonance (*T_d_* > 1) vibration. Under the condition *dT_d_*/*dr* = 0 in Equation (3), it is possible to determine the frequency ratio *r*_0_, at which the displacement transmissibility reaches a maximum value [25]:

(6) $$r_{0}=\frac{\sqrt{\sqrt{1+8\zeta^{2}}-1}}{2\zeta }$$

It is apparent from Equation (6) that the local extreme of the displacement transmissibility (*T_dmax_*) is generally shifted to lower values of the frequency ratio *r* with an increase in the damping ratio *ζ*.

The mechanical vibration damping testing of the investigated rubber composites was performed by the forced oscillation method. The displacement transmissibility *T_d_* was experimentally measured using a BK 4810 shaker in combination with a BK 3560-B-030 signal pulse multi-analyzer and a BK 2706 power amplifier (Brüel & Kjær, Nærum, Denmark) in the frequency range of 2–1500 Hz. Harmonic sine waves were generated by the shaker. The displacement transmissibility was determined from Equation (3) based on the acceleration amplitudes *a*_1_ and *a*_2_ on the input and output sides of the investigated samples by means of BK 4393 accelerometers (Brüel & Kjær, Nærum, Denmark). Measurements of the displacement transmissibility were performed for different inertial masses *m* (i.e., 0, 90 and 500 g), which were located on the upper side of the harmonically loaded investigated samples. Furthermore, vibration damping testing of the investigated rubber samples with a ground plane dimension of 60 mm × 60 mm was performed for three different sample thicknesses (i.e., 4, 8 and 12 mm). Each measurement was repeated five times at an ambient temperature of 23 °C.

#### 2.2.7. Sound Absorption Measurements 

Sound absorption properties of materials are characterized by the sound absorption coefficient *α* (–), which is defined by the ratio [30]:

(7) $$\alpha =\frac{P_{d}}{P_{i}}$$

where *P_d_* is the dissipated acoustic power and *P_i_* is the incident acoustic power. The ability of a material to absorb sound is generally affected by different factors, namely by excitation frequency of acoustic waves, material thickness, structure, density, temperature, humidity and so on. Sound absorption properties of materials are also characterized by the noise reduction coefficient NRC (−), which considers the excitation frequency influence on the sound absorption coefficient. This coefficient is defined as the average value of the sound absorption coefficients of a given material at the excitation frequencies of 250, 500, 1000 and 2000 Hz [31]:

(8) $$NRC=\frac{\alpha_{250}+\alpha_{500}+\alpha_{1000}+\alpha_{2000}}{4}$$

Sound absorption measurements can also be used to determine the longitudinal elastic coefficient *K* of a powder bed. This coefficient is similar to Young´s modulus of elasticity and is defined by the formula [32,33]:

(9) $$K=c^{2}\cdot \rho_{b}={\left(4\cdot h\cdot f_{p1}\right)}^{2}\cdot \rho_{b}$$

where *c* is the speed of sound of elastic wave propagated through a powder bed, *ρ_b_* is the powder bulk density, *h* is the powder bed height and *f_p_*_1_ is the primary absorption peak frequency.

Frequency dependencies of the normal incidence sound absorption coefficient *α* of the tested rubber samples and the loose silica powder were determined by the transfer function method ISO 10534-2 [34] using a BK 4206 two-microphone impedance tube in combination with a BK 3560-B-030 signal pulse multi-analyzer and a BK 2706 power amplifier (Brüel & Kjær, Nærum, Denmark) in the frequency range of 150–6400 Hz. The transfer function method is based on the partial standing wave principle. In this case, the normal incidence sound absorption coefficient *α* is expressed by the equation [35]:

(10) $$\alpha =1-{\left|\frac{H_{12}-e^{-k_{0}\cdot \left(x_{1}-x_{2}\right)\cdot j}}{e^{k_{0}\cdot \left(x_{1}-x_{2}\right)\cdot j}-H_{12}}.e^{2k_{0}\cdot x_{1}\cdot j}\right|}^{2}$$

where *H*_12_ is the complex acoustic transfer function, *k*_0_ is the wave number, *x*_1_ and *x*_2_ are the distances of two microphone positions from the reference plane (*x* = 0) and *j* is the imaginary unit. The complex acoustic transfer function is defined as follows:

(11) $$H_{12}=\frac{p_{2}}{p_{1}}=\frac{e^{k_{0}\cdot x_{2}\cdot j}+r\cdot e^{-k_{0}\cdot x_{2}\cdot j}}{e^{k_{0}\cdot x_{1}\cdot j}+r\cdot e^{-k_{0}\cdot x_{1}\cdot j}}$$

where *p*_1_ and *p*_2_ are the complex acoustic pressures at two microphone positions. Sound damping properties of the tested rubber samples and the silica powder bed were investigated in the impedance tube having inside diameter of 30 mm for different sample thicknesses (i.e., 4, 8 and 12 mm) of the studied rubber composites and various silica powder bed heights (ranging from 10 to 40 mm). Experimental measurements of the sound absorption coefficient were realized at an ambient temperature of 22 °C.

#### 2.2.8. Thermal Conductivity

Experimental measurements of the thermal conductivity over the range of 0.01–10 W·m^−1^·K^−1^ of the rubber composites were carried out according to ČSN 72 1105 standard using a C-Therm TCi thermal conductivity analyzer (C-Therm Technologies, Frederiction, NB, Canada). Average values of the thermal conductivity and their standard deviations were subsequently determined from five measured values.

#### 2.2.9. Thermogravimetric Analysis

Thermogravimetric properties of the investigated cellular rubber samples were realized on a thermal analyzer DTG/60 (Shimadzu, Japan). Experimental measurements were performed at a heating rate of 10 °C/min under nitrogen atmosphere (50 mL/min) in the temperature range from 30 to 500 °C. Thermogravimetric analysis, which consisted in measurements of the weight loss ∆*m* of the tested rubber samples, was evaluated using ta60 Version 1.40 (Shimadzu, Japan) software. The measured results were performed in triplicate.

## 3. Results and Discussion

### 3.1. Curing Characteristics

**Figure 1:**
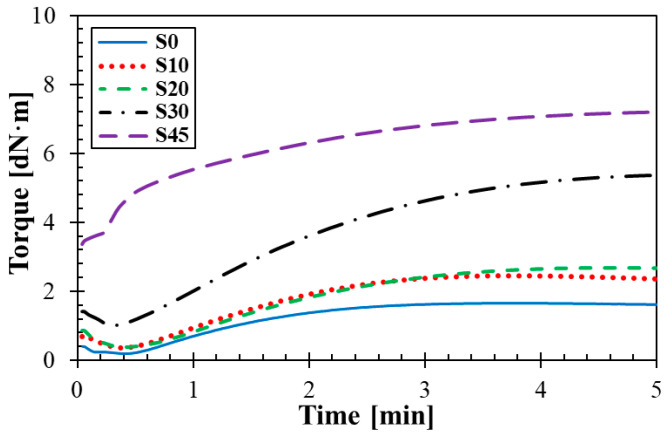
Time dependencies of vulcanization characteristics.

Curing curves of the tested rubber composites are demonstrated in Figure 1. Curing parameters of these materials are shown in Table 2. It is visible (see Figure 1) that the minimum (*M_L_*) and maximum (*M_H_*) torques increase with an increase in the silica concentration due to a higher mechanical stiffness of the rubber composites at higher silica concentrations. Similarly, the optimum cure time *t*_90_ increases with increasing the silica concentration. Table 2 also shows the scorch time *t_s_*_1_, which is characterized by the fact that the rubber composite was still plastic during this time. Therefore, the blowing process of the tested rubbers should also be carried out during the time *t_s_*_1_. 

### 3.2. Rubber Processing Analysis

**Figure 2:**
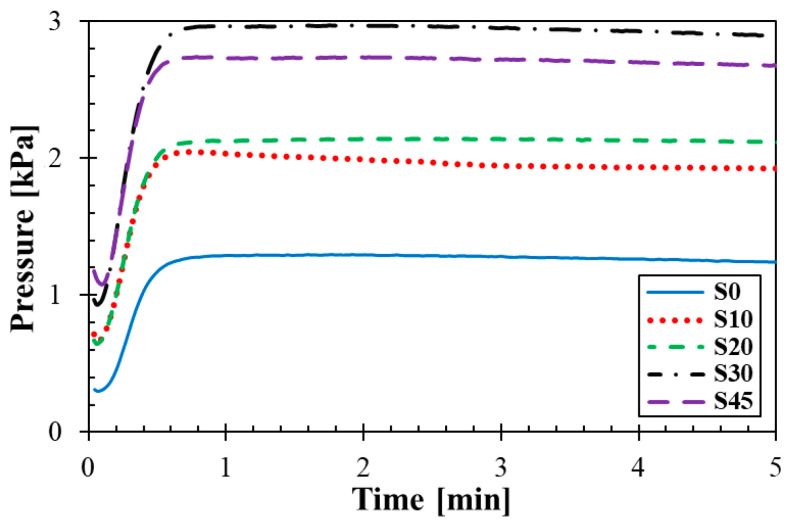
Time dependencies of pressure curves.

**Figure 3:**
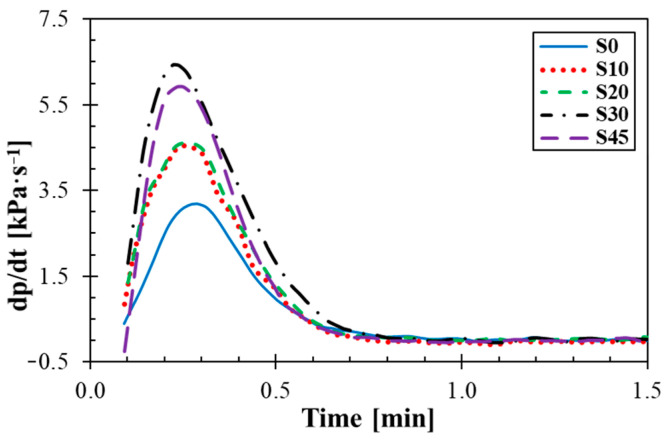
Time dependencies of rate of blowing.

Time dependencies of pressure curves and the rate of blowing of the investigated rubber composites are shown in Figure 2 and Figure 3. It is evident from these figures that the blowing process of the rubbers took place in three phases. In the first phase (i.e., preheating), the rubber composite is heated, its viscosity is reduced to a minimum and the blowing agent was not decomposed. In addition, a skin is formed in order to prevent profile collapse. In the second stage (i.e., expansion), the blowing agent was decomposed with increasing the vulcanization rate. A relative balance between pressure gas and vulcanizate formation created a cell structure. The degree of vulcanization reached its maxima in the third stage (i.e., curing). If the decomposition of the blowing agent starts late, the production of released gas from the decomposition of the blowing agent could be limited during the curing process, which could lead to imperfect cellular structures of rubbers. It is evident from Figure 2 that a rapid increase in pressure (i.e., blowing) took place in the second phase, when the decomposition of the blowing agent occurs only with the increasing rate of vulcanization. As shown in Figure 3, the fastest decomposition of the blowing agent occurred at the maximum rate of blowing. The vulcanization and blowing processes of the rubber composite containing 45 phr of silica were more difficult due to higher filler content. These processes were slower because the silica filler bound the vulcanization system due to the content of polar groups. Therefore, the rubber composite containing 45 phr of silica became more viscous during the blowing process, and thus the blowing of this rubber composite was imperfect.

### 3.3. Optical Microscopy

**Figure 4:**
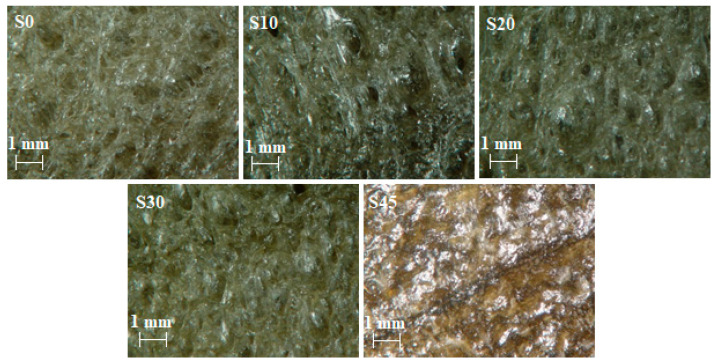
Optical microscopy images of the studied rubber samples.

As can be seen from the optical microscopy images (see Figure 4), it is possible to observe distinct cavities caused by evaporation of the blowing agent. It is evident that the most distinct cavities are observed for the rubber composite containing 0 phr of silica. Smaller cavities are observable in the rubber samples containing 10, 20 and 30 phr of silica. The size of these cavities is still sufficient to ensure good thermal insulation properties of these rubbers. It is also obvious from Figure 4 that there was practically no cellular structure of the rubber composite containing 45 phr of silica. This is due to the fact that silica is reinforcing filler, which increases the viscosity of rubber composites at high silica concentrations. Therefore, sufficiently large cavities were not created during the blowing process in the rubber composite with 45 phr of silica. This fact also corresponds with the time dependence of the rate of blowing (see Figure 3), where the maximum rate of blowing for the rubber composite containing 45 phr of silica was lower compared to the maximum rate of blowing of the rubber composite with 30 phr of silica. For this reason, the rubber composite containing 45 phr of silica was not suitable in terms of thermal insulation properties.

### 3.4. Mechanical Properties

#### 3.4.1. Rebound Resilience

The results of the average values of the rebound resilience and their standard deviations are shown in Table 3. The rebound resilience decreased from 51% to 35% for S0 and S45 rubber composites, respectively. It can be concluded that higher silica concentrations in rubber composites led to a higher ability to transform mechanical energy into heat, and thus to a decrease in the rebound resilience.

#### 3.4.2. Compression Testing

**Figure 5:**
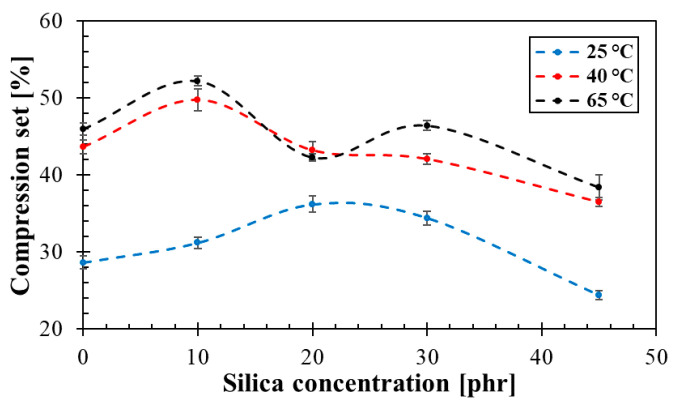
Effect of silica concentration on compression set of the investigated rubber composites under different compression temperatures.

Dependencies of permanent deformation vs. silica concentration for three different temperatures (i.e., 25, 40 and 65 °C) are demonstrated in Figure 5. It can be observed that the compression set generally increased with increasing temperature. It is also evident that the permanent deformation decreased at higher silica concentrations. This was due to the higher silica content in rubber composites and the formation of smaller cavities that were able to return the rubber composites to their original state [24]. The highest compression set values (i.e., 52.2 and 48.7%) at the given compression temperatures (i.e., 65 and 40 °C) were obtained for the rubber composites containing 10 phr of silica. At the compression temperature of 25 °C, the highest compression set of 36.2% was found for the rubber composite containing 20 phr of silica. 

#### 3.4.3. Mechanical Vibration Damping Testing

**Figure 6:**
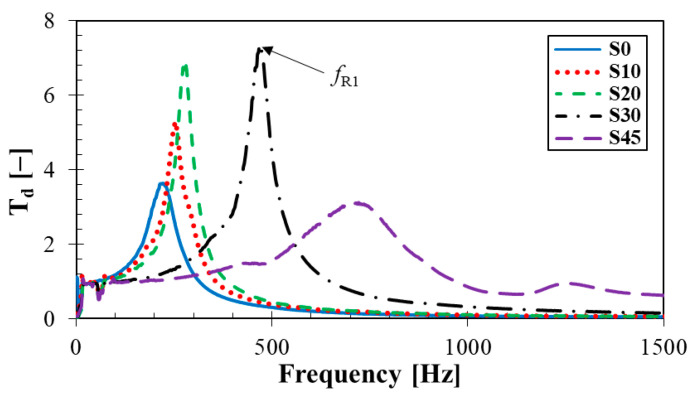
Effect of silica concentration on frequency dependencies of the displacement transmissibility of the tested rubber composites measuring *t* = 4 mm in thickness and loaded with inertial mass *m* = 90 g.

Frequency dependencies of the displacement transmissibility of the tested rubber composites measuring *t* = 4 mm in thickness and containing different concentrations of silica are shown in Figure 6. In addition, the rubber samples were loaded with an inertial mass of 90 g. It is evident from this comparison that the concentration of silica had a significant influence on vibration damping properties of the tested composite materials. The ability of a material to damp mechanical vibration is generally decreasing with increasing the concentration of silica, which was in accordance with measurements of the rebound resilience and the permanent deformation. It was again due to higher stiffness *k* (or lower damping ratio *ζ*) of the rubber composites, which were produced with higher concentrations of silica. These facts resulted in a lower transformation of input mechanical energy into heat during forced oscillations [36], and in an increase in the values of the damped and undamped natural frequencies (see Equation (6)) [37]. Therefore, the first resonance frequency (*f_R_*_1_) value was shifted to the right (see Figure 6) with increasing concentration of silica, namely from 218 Hz (i.e., sample S0) to 721 Hz (i.e., sample S45), as indicated in Table 4. 

The vibration damping properties of the investigated harmonically loaded rubber samples were also influenced by their thickness *t*, the inertial mass *m* and the excitation frequency *f*. 

**Figure 7:**
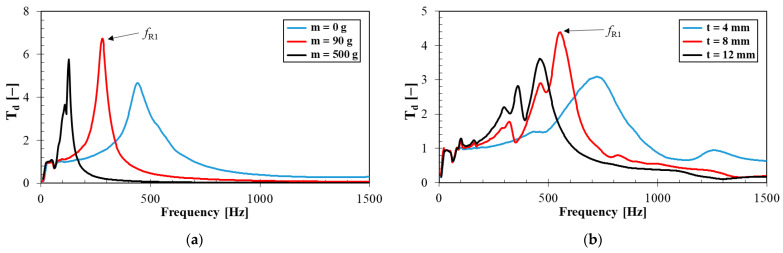
Frequency dependencies of the displacement transmissibility: (**a**) Rubber samples containing 20 phr of silica measuring *t* = 4 mm in thickness and loaded with different inertial masses; (**b**) rubber samples of various thicknesses containing 45 phr of silica and loaded with inertial mass *m* = 90 g.

The effect of the inertial mass on vibration damping properties for the S20 rubber type measuring *t* = 4 mm in thickness is shown in Figure 7a. It is visible that better vibration damping properties were generally observed for higher inertial masses *m*. For this reason, the inertial mass had a positive influence on mechanical vibration damping, which is reflected in a shift of the first resonance frequency peak position to lower excitation frequencies, i.e., in the decrease of the frequency *f_R_*_1_ (see Table 4) from 434 Hz (*m* = 0 g) to 120 Hz (*m* = 500 g). This finding is consistent with Equations (4) and (6). It should be noted that the magnitude of inertial mass is limited only to the range of elastic deformations of the harmonically loaded rubber specimens.

The effect of the sample thickness on vibration damping properties is shown in Figure 7b for the S45 rubber type loaded by an inertial mass of 90 g. It is visible that a higher material thickness led to lower values of the frequency *f_R_*_1_, i.e., from 721 Hz (*t* = 4 mm) to 457 Hz (*t* = 12 mm), as indicated in Table 4. For this reason, the rubber thickness generally has a positive effect on vibration damping and leads to a higher transformation of input mechanical energy into heat during dynamic loading. 

It is also visible from Figure 6 and Figure 7 that the vibration damping properties were also significantly influenced by the excitation frequency *f*. It is evident from these frequency dependencies that the resonant mechanical vibration (i.e., *T_d_* > 1) was achieved at low excitation frequencies depending on the rubber composite type, its thickness *t* and the inertial mass *m*. For example, for the S45 rubber type measuring 4 mm in thickness and without inertial mass (i.e., *m* = 0 g), the resonant mechanical vibration was observed over the entire frequency range. In the case of the S0 rubber type measuring 12 mm in thickness and loaded with the highest inertial mass (i.e., *m* = 500 g), the resonant mechanical vibration was observed at significantly lower excitation frequencies (namely at *f* < 86 Hz). On the contrary, the damped mechanical vibration (i.e., *T_d_* < 1) was generally obtained at higher excitation frequencies (see Figure 6 and Figure 7).

### 3.5. Sound Absorption Properties

**Figure 8:**
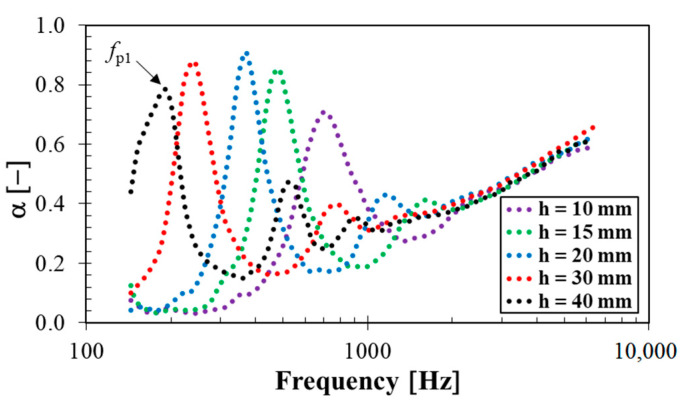
Frequency dependencies of the sound absorption coefficient of the silica powder for different loose powder bed heights.

Frequency dependencies of the sound absorption coefficient of the loose silica powder depending on its height are shown in Figure 8. The obtained results of acoustical and mechanical quantities from sound absorption measurements are shown in Table 5. It is clear from the calculated values of the noise reduction coefficient NRC (see Equation (8)) that sound damping properties of the tested loose silica filler generally increased with increasing the powder bed height. As is shown in Figure 8, sound absorption properties of the silica powder were similar at higher excitation frequencies (i.e., at *f* > 2 kHz) independently of the powder bed height. It is also visible that the maximum value of the sound absorption coefficient, which is proportional to the primary absorption peak frequency *f_p_*_1_ (i.e., *α_max_* = *α_fp_*_1_), generally shifted towards lower excitation frequencies with increasing the powder bed height (see Figure 8). The speed of sound *c* and the longitudinal elastic coefficient *K* were subsequently determined from Equation (9). It was found that a higher powder bed height led to a higher speed of sound *c*, and thus, to higher values of the longitudinal elastic coefficient *K*. For this reason, the mechanical stiffness of the studied silica filler generally increased with increasing the silica bed height *h*.

**Figure 9:**
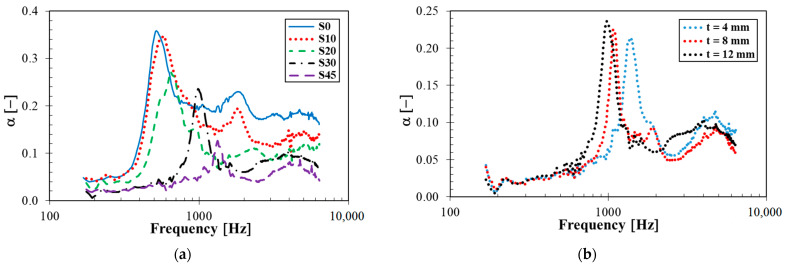
Frequency dependencies of the sound absorption coefficient: (**a**) Rubber samples measuring *t* = 12 mm in thickness at different concentrations of silica filler; (**b**) rubber samples of various thicknesses with 30 phr of silica filler.

Frequency dependencies of the sound absorption coefficient of the tested rubber composites measuring *t* = 12 mm in thickness with different concentrations of silica are shown in Figure 9a. It is evident that sound absorption properties generally increased with decreasing silica concentrations. This fact was influenced by the rubber stiffness, which increases with increasing the silica concentration, as well as the longitudinal elastic coefficient of the loose powder beds (see Table 5). For this reason, better sound damping properties were obtained for rubber samples containing lower silica concentrations, at which the rubber samples exhibit lower stiffness and their structure is more spongy-like. This finding is consistent with the fact that porous, spongy and fiber materials belong to suitable materials in order to absorb sound [38]. The above-mentioned phenomena were also in good agreement with the vibration damping measurements, when a better ability of damp mechanical vibrations was generally obtained in rubber composites containing lower concentrations of silica. 

It was also found that sound damping properties increase with increasing the sample thickness (see Figure 9b), especially at lower excitation frequencies. This finding was confirmed on the basis of the noise reduction coefficient. As indicated in Table 6, the noise reduction coefficient generally increased with an increase in rubber thickness and the decreasing concentration of silica. Its value ranges from 0.028 (S45, *t* = 4 mm) to 0.182 (S0, *t* = 12 mm). For these reasons, sound absorption properties of the investigated rubber composites were relatively low. 

### 3.6. Thermal Properties

#### 3.6.1. Thermal Conductivity

**Figure 10:**
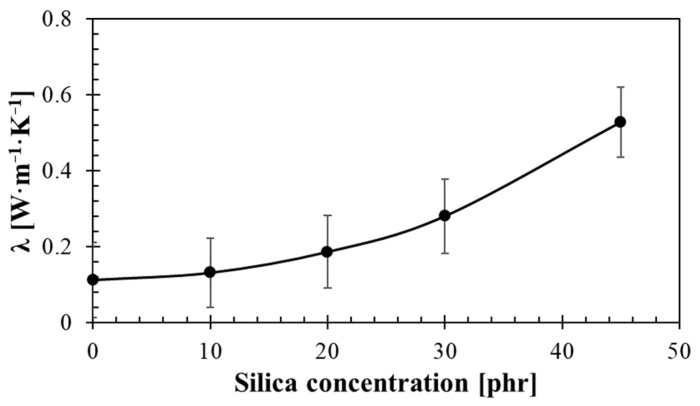
Effect of silica concentration on thermal conductivity of the tested rubber composites.

The effect of silica concentration on thermal conductivity of the studied rubber composites is shown in Figure 10. Observed data show an exponential increase in thermal conductivity with the increasing silica concentration. Low silica content led to larger pore sizes in rubber composites, which is accompanied by lower values of thermal conductivity. Therefore, rubber composites containing pores of larger sizes can be used as thermal insulators. As shown in Figure 10, a significant increase in thermal conductivity was achieved at higher concentrations (>30 phr) of silica. In these cases, the rubber composite is too stiff and viscous, which prevented the formation of larger pores. The thermal conductivity of 0.508 W·m^−1^·K^−1^ for the rubber composite containing 45 phr of silica was approximately double compared to the thermal conductivity of the rubber composite with 30 phr of silica. For this reason, a porous structure of the rubber composite containing 45 phr of silica was practically not formed, which led to a significant decrease in thermal insulation properties of rubber composites containing high concentrations (i.e., over 45 phr) of silica. 

#### 3.6.2. Thermogravimetric Analysis

**Figure 11:**
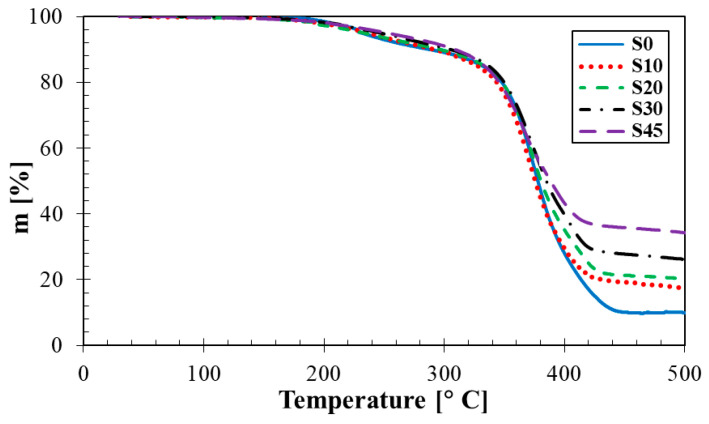
Thermogravimetric analysis results of the studied silica/rubber composites.

As illustrated in Figure 11, the rubber composites were characterized by a two-step symmetric degradation of similar curve shapes, regardless of filler amount. The first declination on thermogravimetric curves started above 150 °C and continued until 300 °C with the corresponding weight loss of about 10% (*w*/*w*). Exceeding this temperature limit (i.e., about 300 °C), a rapid degradation occurred, and was accompanied by a noticeable decrease in the sample weight. At the temperatures from 420 to 440 °C, the degradation process stopped, while the degradation extent was apparently dependent on the filler concentration. 

As is obvious from Table 7, higher reduction in the weight loss ∆*m* was generally observed at lower filler concentrations. It was found that the rubber sample without any silica filler (i.e., 0 phr) exhibited the weight loss of 90.42% (*w*/*w*) in the entire temperature range. On the other hand, the rubber sample containing the highest filler concentration (i.e., 45 phr) showed the highest thermal stability at the weight loss ∆*m* = 66.63% (*w*/*w*). Therefore, higher concentrations of the silica filler generally led to a higher thermal stability of the investigated rubber composites.

## 4. Conclusions

Rubber composites are widely applied in many areas at the present time. It is often necessary to optimize rubber properties for given purposes. This research was focused on the study of mechanical, sound absorption and thermal properties of cellular rubber composites that were filled with different concentrations of silica nanofiller. Nevertheless, the tested rubber composites were produced with the same blowing agent concentration. In addition, the production of the rubber samples was carried out according to a new methodology in order to optimize the blowing and vulcanization processes of these samples. Based on this research, it can be concluded that the concentration of silica filler in the rubber composites had a significant influence on the mechanical stiffness of the rubbers, and thus on their sound absorption and thermal properties.

The mechanical properties examined included rebound resilience, compression testing and mechanical vibration damping properties of the investigated rubber composites. It was found that the mechanical stiffness of the rubber composites generally increased with increasing concentration of silica filler. The rebound resilience of the rubber composite containing 45 phr of silica was approximately 32% lower compared to the rubber composite containing 0 phr of silica. Similarly, the compression set of the rubber composites with 45 phr of silica was approximately 16% lower compared to those of the rubber composites with 0 phr of silica. These findings were in good agreement with vibration damping measurements that were performed using the non-destructive forced oscillation method based on the displacement transmissibility. It can be stated that the first resonance frequency peak position increased with an increase in mechanical stiffness leading to lower vibration damping properties of the rubber composites. Therefore, a higher concentration of silica filler in rubber composites generally led to a lower transformation of input mechanical energy into heat under dynamic loading of these rubbers. The mechanical properties of the investigated rubber composites were also in agreement with their sound absorption properties. It was found based on the sound damping measurements that higher concentrations of the loose silica powder led to higher values of the longitudinal elastic coefficient, and thus to a higher mechanical stiffness of the silica filler. Furthermore, sound absorption properties of the rubber composites generally decreased with increasing silica concentration. For this reason, a higher ability to damp sound was found for the rubber composites of more spongy-like structure, which is typical for rubbers containing lower concentrations of silica filler. 

It was also found that higher concentrations of silica nanofiller generally led to a decrease in thermal degradation and an increase in thermal conductivity of the investigated rubber composites. Therefore, in practice, rubber composites containing lower concentrations of silica filler can be used as thermal insulators.

From the experimental data, it can be concluded that the mechanical stiffness of the tested silica-filled rubber composites can be also evaluated by the non-conventional methods of mechanical vibration damping and sound absorption. These methods are relatively simple, inexpensive, fast and non-destructive, which are their undeniable advantages compared to conventional methods used to determine the mechanical stiffness. 

The investigated silica-filled rubber composites can be used in applications where higher shape recovery, stiffness-to-weight ratio and thermal conductivity are required, e.g., in the manufacture of rubber components in automotive, aerospace and footwear industries.

## Figures and Tables

**Table 1:** Recipe of the investigated rubber compounds.

Ingredients	Rubber Designation				
	S0	S10	S20	S30	S45
	Loading (phr)				
SMR–20	100	100	100	100	100
Silika KS–408	0	10	20	30	45
ZnO	2	2	2	2	2
Vulkanox 4020	1	1	1	1	1
Porofor ADC	5	5	5	5	5
Nyflex 228	10	10	10	10	10
Stearic acid	1	1	1	1	1
TBBS	1	1	1	1	1
Sulphur OT33	2	2	2	2	2

**Table 2:** Curing parameters of the studied rubber composites.

Rubber Type	*M_H_* [dN·m]	*M_L_* [dN·m]	*t_s_*_1_ [min]	*t*_90_ [min]
S0	1.67	0.20	0.54	1.76
S10	2.45	0.37	0.57	1.73
S20	2.67	0.41	1.09	2.31
S30	5.28	1.03	0.87	3.36
S45	7.14	3.37	0.31	3.54

**Table 3:** Values of rebound resilience of the tested rubber composites.

Rubber Type	Resilience [%]
S0	51 ± 1
S10	48 ± 1
S20	44 ± 1
S30	41 ± 1
S45	35 ± 1

**Table 4:** First resonance frequency (*f_R_*_1_) in Hz of the studied rubber composites as induced by harmonic force vibration depending on rubber thickness (*t*) and inertial mass (*m*).

Rubber Type	*t* [mm]	*m* [g]		
		0	90	500
S0	4	399 ± 13	218 ± 10	100 ± 5
	8	192 ± 8	115 ± 5	62 ± 3
	12	176 ± 6	80 ± 3	55 ± 2
S10	4	417 ± 15	251 ± 11	106 ± 4
	8	235 ± 10	122 ± 4	70 ± 3
	12	220 ± 9	103 ± 4	59 ± 2
S20	4	434 ± 14	279 ± 12	120 ± 5
	8	294 ± 12	178 ± 6	77 ± 3
	12	257 ± 11	146 ± 4	73 ± 3
S30	4	525 ± 15	468 ± 18	196 ± 6
	8	436 ± 16	343 ± 11	140 ± 4
	12	396 ± 13	278 ± 9	119 ± 4
S45	4	1239 ± 25	721 ± 20	284 ± 9
	8	804 ± 19	548 ± 15	201 ± 7
	12	545 ± 14	457 ± 12	154 ± 5

**Table 5:** Results of measured and calculated acoustical and mechanical quantities for different loose powder bed heights of the applied silica filler.

Quantity	*h* [mm]				
	10	15	20	30	40
NRC [–]	0.259	0.359	0.302	0.458	0.384
*f_p_*_1_ [Hz]	696	480	364	244	184
*α_fp_*_1_ [–]	0.709	0.854	0.911	0.879	0.784
*c* [m·s^−1^]	27.8	28.8	29.1	29.3	29.4
*K* [MPa]	0.132	0.141	0.144	0.146	0.147

**Table 6:** Values of noise reduction coefficient (NRC) of the studied rubber composites.

Rubber Type	*t* [mm]		
	4	8	12
S0	0.086	0.113	0.182
S10	0.066	0.107	0.158
S20	0.065	0.098	0.110
S30	0.047	0.067	0.084
S45	0.028	0.030	0.037

**Table 7:** Values of weight loss (∆*m*) of the investigated rubber composites containing different silica concentrations in the temperature range of 30 to 500 °C as the results of thermogravimetric analysis.

Rubber Type	∆*m* [% *w*/*w*]
S0	90.42 ± 0.40
S10	83.29 ± 0.53
S20	79.34 ± 0.43
S30	73.33 ± 0.58
S45	66.63 ± 0.83

## Data Availability

Not applicable.
